# Technology and Older Women: Considerations Regarding Their Use and Misuse

**DOI:** 10.3389/fpubh.2022.853983

**Published:** 2022-06-29

**Authors:** Chyrisse Heine, Susan Feldman

**Affiliations:** School of Health, Ballarat, Federation University Australia, Mount Helen, VIC, Australia

**Keywords:** technologies, older women, challenges, ethics, dignity, ageism, self-determination, privacy

## Abstract

Health and wellbeing are inextricably linked to an individual's capacity for continued meaningful engagement and connection with the world around them. Technological innovations designed to maximize the quality of life for older women range from sophisticated bio-medical interventions to ordinary day-to-day communication devices. Many innovations can ensure a higher quality of life for older women and support and care as required.

In this article, we consider: (1) The range of appropriate technologies currently available for older women, their families and communities. (2) The way technology contributes to the maintenance of optimum physical health and wellbeing for older women. (3) The significant challenges and considerations associated with the incorporation of technologies into their daily lives.

## Introduction

Estimates are that the world's population of people aged 60 years and over will increase to nearly 1.5 billion by 2050 (1). As of June 2020, more than half of older Australians were aged 65–74 years (56%), 3 in 10 were aged 75–84 years (31%) and around 1 in 8 were aged 85 years and over (13%) (2). The increase in longevity globally is reflected in Australian population trends with women currently outliving their male counterparts by up to 4.1 years in 2018–2020 (2, 3).

Living a long time can be a challenge for any individual, especially those who strive to achieve a healthy older age. Thus, it is useful to draw upon concepts of successful or healthy aging which specifically acknowledges aging as a dynamic process across the life span (4). Increasingly, older Australian women face the challenge of divorce, separation or widowhood. Many women choose to continue living in their own homes and outside of institutional care arrangements for as long as is possible, despite the physical, cognitive or mental health changes that may accompany growing older (5).

## Context

In line with both international and Australian policies to provide support and care for individuals to “age in place” (6–8), significant numbers of older women continue to live independently (5, 9).

The weeks and months of “lockdown” isolation and separation during the global COVID-19 pandemic were, for many older women, confronting, particularly for individuals living alone. It was a time of disruption of supportive and reciprocal relationships with family, community, and peers (10). For some older women, communication devices and social media platforms may have facilitated a certain level of connection and positive exchanges with family, friends, informal networks, and activities (11, 12). In the short term, technology might have eased the sense of dislocation and loneliness that can disrupt a person's sense of inclusion, their psychosocial health and wellbeing (13).

## Enabling and Assistive Technologies

Daly et al. (14) describe technology in the broadest sense, as encompassing devices which “range from simple tools such as glasses for weak eyesight, to large technological systems that govern our hospitals and health care systems” (pxxi). “Enabling and assistive technologies” describe the range of devices and tools that promote and assist with the maintenance of optimum health and wellbeing and are pertinent at an individual, family, community and societal level regardless of gender, age or health status (15). These range from voice activated devices and home systems (e.g., Automatic lighting, stove-top monitors), mobile telephones, computers (e.g., Screen readers and speech and voice recognition), email, social media to face-to-face communication platforms, including telehealth (16). These applications can contribute to the overall social connectedness and thus quality of life, of older women, especially those who live alone or with minimal support (13).

We scanned the peer-review literature to review appropriate models that comprehensively illustrate the application of technology in a wide range of domains. This search indicated that there is a dearth of information presenting gender specific models of technology and in particular relating to older women see Table 1. These innovative medical technologies, that have a critical life-saving role include personalized health monitoring devices, robotics and mechanical protheses, especially in the surging field of regenerative medicine (19). Technologies for personal safety and peace of mind for both individuals and their family members, extend to the installation of home and personal devices that detect and alert and vehicle safety devices. These may be related to falls prevention, smoke alarms and temperature controls, wandering management and door locking systems and vehicle safety devices such as automatic brake engagement and lane detection technology (20). Medication compliance technologies and cognition assessment tool, video monitoring, and mobile phones are not only used for communicating but for tracking and locating individuals.

**Table 1:** Selected technological frameworks relevant to maintaining the health and wellbeing of older Women's lives.

	**Alwan et al. (17);** **Resnick et al. (18)**	**Roco et al. (26)**	**Coughlin et al. (20)**
Safety	• Fall detection and prevention • Stove use detectors • Smoke/temperature detectors • Door locks • Wander management systems		“Monitor” - monitoring of health and safety using intelligent devices, appliances, robotics internet (e.g., Status of the vehicle driver) enabled services and predictive behavior models. e.g., Corrected vehicle positioning to prevent off-road excursions
Health and wellbeing	• Telemedicine and tele-health technologies, (e.g., Ambulatory and wearable monitors, video phones and two-way video stations) • Medication compliance • Cognitive assessment (stimulation and entertainment systems and assessment and reminder systems)	• Self-delivered nano-medical intervention • Self-monitoring of physiological wellbeing and dysfunction using nano implant devices (e.g., metabolic and anatomical monitoring to track energy balance) • Nanobiotechnologies for adjusting organ performance and to aid localized drug or metabolite delivery to artificial organs	“Manage” – vehicle software including adaptive cruise control (ACC), blind-spot detection, parking assistance, rumble strips, lane markings, lane departure prevention, and smart airbags
Social connectedness/ communication	• Senior friendly email and web portal systems • Video phones and two way video conferencing	• Multi modalities for living and hearing impaired (e.g., different modes of communication including talking environments and 3-D touch screens to enable access to the Internet • Information systems designed to present medical data in ways intelligible to laypersons	• “Motivate” – creating a more comprehensive and cohesive connection between the driver-vehicle unit and developing IntelliDrive and intelligent transportation system (ITS) applications. • Information supports the display of important feedback to the driver and can trigger appropriate alerting or calming features aimed at refreshing and reducing stress, and thus improving safety • Feedback systems that communicate without startling the driver
Mobility	Mobility aids traditionally used to enhance balance and/or help in weight support adapted and enhanced to allow seniors to navigate their environments safely	• Nanobiotechnology (e.g., improved joint replacement) • Driving: computers and sensors driven by nanotechnology combined with on-board artificial intelligence helping the driver plan routes and avoid hazards • Nanobiotechnology: on-board biosensors to monitor driver stress and physiological condition, to be fed back to the car's computer • Implanted devices to improve cognizance and keep driver alert	Driving (e.g., improved accessibility through design and information services)

As can be seen from Table 1, increasingly technological enhancements have a significant role in maximizing the mobility, safety and independence of older women, especially when moving out and about in the community (20).

## Challenges

Community acceptance and uptake of innovative technologies might not be unproblematic and without criticism, particularly from those individuals and communities who hold specific philosophical, cultural or religious beliefs. Consideration must also be given to the confidence or willingness of older women and their carers to engage with new technological developments (8, 21, 22). Furthermore, women living beyond urban areas face the challenges associated with limited access to technology either through issues of affordability, unreliable connections, or in some instances especially in regional and rural areas, lack of internet services altogether.

## Illustrative Vignettes

The following vignettes illustrate the role of ever evolving technology in optimizing health, encouraging independent living and promoting a sense of autonomy and control for older women who continue to “age in place” at home (7, 8).

### Scenario 1 – “a Visit to the GP”

Edie is aged 86 years and lives alone in her own home. Following heart surgery, she attends a post-operative appointment with her General Practitioner (GP). Edie is also diabetic. Her family are concerned about her ability to drive and live alone. Prior to the consultation, and with Edie's consent, the GP's practice manager downloaded and collated electronically all of Edie's tests and interventions, current medications, ECG and EEG results via e-record sharing between laboratories, pharmacy and GP. Alford and Johnston (19) remind us that telehealth relies on health services that have access to reliable electronic patient record systems with permission to share patient records across the health service network. Edie's case illustrates technological innovations that assisted her GP to access the Australian national e-health networking systems (My HealthRecord) via a National Highspeed Broadband Network (NBN). The GP recommends that Edie: consults an audiologist for fitting of a digital hearing aid with state-of-art technology such as speech enhancement and directional microphone to improve her declining hearing due to presbycusis; considers using a personal button necklace security alarm, a video camera system at home as well as a blood pressure monitoring system including a wrist-watch to alert to irregular breathing or pulse rates, and vehicle mats to reassure Edie and her family about her driving safety.

### Scenario 2 – “Safe and Sound at Home”

Rose is aged 97 years, recipient of a part government Pension and despite changes of aging, lives alone in her own home with limited help. “Meals on wheels” delivers food once a week, supplemented by supermarket deliveries and food prepared by family. Rose's home has access to NBN and she is “iPad savvy,” which enables online shopping once the refrigerator system has alerted her to the need for replenishment. Keeping in regular contact with family is via email, text and Skype. Technologies enable her TV to be set at a predetermined volume, and food be heated safely on a pre-set stove. Rose feels quite safe and secure in her home where she spends time in her small garden which is fitted with an automatic watering system. Rose also enjoys walking and meeting up with neighbors (using GPS).

Technologies have assisted Rose in retaining her sense of security, independence and autonomy. Other innovations include automated home security, electronic warning devices that monitor and shut down heating and cooking equipment when danger is detected, and automatic climate control. In addition, Rose communicates with family and friends with local government sponsored tele and phone links and high-speed internet systems. Importantly, both Edie and Rose were assured that protecting their privacy was of tantamount importance regardless of the array of technological devices introduced into their homes.

In summary, each vignette has illustrated those technological innovations and devices that can enhance an older woman's health and wellbeing. This is particularly so through active participation in family, community and social life. The two examples have also included examples of technologies and devices that can assist an older woman to achieve a sense of autonomy and control over their own life, despite their changing health or physical capacities.

**Figure 1:**
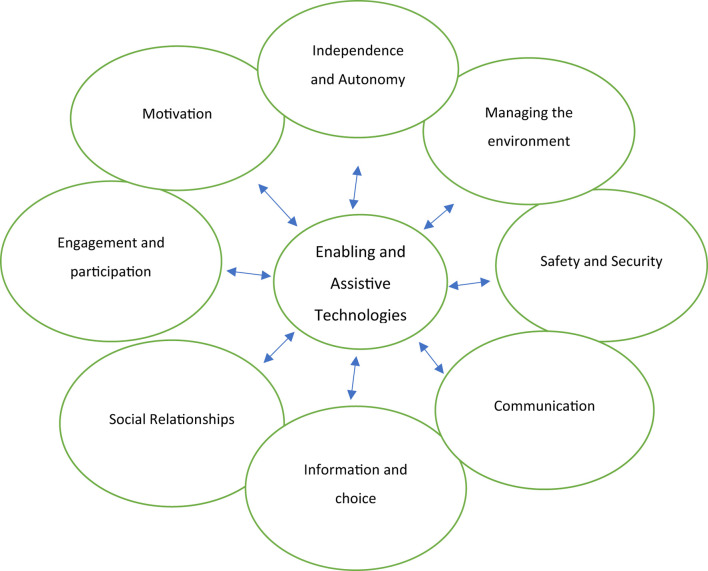
Technology in the promotion of health and wellbeing in older women.

Figure 1 is an illustration of the overarching and inextricably linked key elements essential to any older woman's health and wellbeing. While they are distinct one from the other, they are also inextricably interrelated one to the other with one impacting the other in different ways and at different times in a woman's life span.

## Discussion

The universal uptake of any technological innovation, particularly those which relate to health must be tempered with ongoing critical analysis and evaluation of the proposed health benefits as well as the potential for negative consequences or risks.

### Ethical Considerations

Any discussion regarding the role of technologies should consider whether there may be a conflict between prevailing community standards and moral rules, or principles related to their use. We assert that any discussions about the potential role of technology in the lives of older women must reflect contemporary ethical standards. In addition, ethical considerations should not only apply to clinical, medical or social settings, but equally transcend into the policy, planning and practice arenas (23). Charlesworth (24) suggests that the issues and problems related to medical ethics or bioethics are both diffuse and complex.

### Dignity

The right of any individual for self-determination and choice underpins the idea of the protection of human dignity and the respect of older people is central to the WHO-UN policy framework of Healthy Aging (25). Contemporary medical diagnosis and treatment is aided by the latest laboratory testing of human functioning, along with a range of readily available and relatively affordable physical interventions and life-changing procedures such as hip and knee replacements or repair and replacement of heart vessels and valves. The value of these technological interventions, however, must always be measured against individual circumstances and wishes. Questions must be raised about whether the outcomes of any procedures are necessarily positive for the dignity of the individual (23). By way of example, the use of a hearing aid may be perceived by some individuals as an indicator of aging and accompanying loss of dignity, no matter how advanced the technology.

The convergence of technologies not only brings the possibility to improve every dimension of human life, but also a warning about the problematic nature of striving to improve human performance or an unreasonable effort to extend life (26). For example, it is possible that an older woman who has outlived her partner may not wish to have the latest technology such as a pacemaker to improve or extend her quality of life. Such procedures alone cannot resolve her experience of loneliness, loss and grief.

### Ageism

It is the case that ageist attitudes continue to abound (25, 27, 28). Older women and men are very often viewed as vulnerable, dependent and a potential burden on community resources without the capacity to represent themselves in specific negotiations, particularly around management of their health and wellbeing. The consequence of stereotyping older people and their ability to make important decisions has the potential for “technological paternalism where the healthcare system acts to control people in their perceived best interests, perhaps without their consent” [(15), p. 38] and highlights further questions about the damage to individuals with the presumption of incompetence and decrepitude. Unfortunately, there is still a common and incorrect view that as women transition to older age they are automatically dependent and passive recipients of services and care (25, 29).

Of central concern to many older women is being able to maintain their relevance, influence and inclusion as active participants in their own lives. This they indicate, can only be achieved through proactive engagement in making informed choices about the direction of their own lives (29, 30).

### Self-Determination and Autonomy

Moody (31) suggests that “few principles of contemporary bioethics are as honored as the ideal of autonomy” (p. 134). Decisions about the choice and uptake of aids and devices that enable an individual woman to continue living in her own home can be complex, especially since the individual must be involved in a process of “negotiated consent” (p. 136). As much as is practicable, older women must be provided with relevant and up to date information so that they are involved in all decision-making process about whether the proposed technologies are appropriate for them. The inclusion of older women in this process is also central to ensuring that the uptake is successful, thus, maintaining a sense of self determination and autonomy in the face of increasing reliance on assistance from others often presents a challenge, especially so for older women.

### Privacy

All individuals value the concept of privacy and older women are no exception. A fine balance however must be struck between privacy, personal risk, and the integration within a home setting of a range of monitoring technologies. Unfortunately, home surveillance devices can also be intrusive and compromise older women's privacy and sense of self-determination. In addition, regardless of the good intent, we must be wary of how, even with consent, these devices can lead to the invasion of an individual's home, their personal space (13, 31). Consideration must be given to whether the social and ethical impacts of enabling technologies reflect contemporary community standards and expectations regarding the protection of an individual's privacy (32). Consequently, it is vital to consider how the access to and control of medical and health information might be compromised especially, within the context of the development of sophisticated technology and information sharing systems across the health and service system.

## Conclusion

It is only by thinking about technologies as constantly changing and evolving entities that we can vigorously and critically examine their increasing power and potential to enhance the quality of life of women as they enter their older years. An array of technologies will enable women to participate in the social world around them, and as best as possible to maximize the independent and autonomous lifestyle of their choice. We therefore envisage that new and emerging technologies will continue to play a key role in ensuring the provision of high-quality support and care as deemed appropriate by older women themselves. It is important to ask ourselves what are the ultimate goals when introducing technology into an older woman's life?

We emphasize that the promotion of any technologies into daily life must ensure that there is as little compromise as is possible in an individual's needs or wishes for privacy regardless of their gender, language, socio-economic status, culture, geographic region, and of course age.

There is no doubt that technologies play a vital role in the enhancement of the psycho-social health, and well-being of older women right now and in the future. We must query: What kind of technological advancement will there be in the future as we grow old? Finally, we question whether the increased dependence on technological devices in the place of personal interaction will lead to a greater proportion of older women without regular human contact and we ask ourselves what are the implications if we follow this precarious path?

## Data Availability Statement

The original contributions presented in the study are included in the article/supplementary material, further inquiries can be directed to the corresponding authors.

## Author Contributions

All authors listed have made a substantial, direct, and intellectual contribution to the work and approved it for publication.

## Conflict of Interest

The authors declare that the research was conducted in the absence of any commercial or financial relationships that could be construed as a potential conflict of interest.

## Publisher's Note

All claims expressed in this article are solely those of the authors and do not necessarily represent those of their affiliated organizations, or those of the publisher, the editors and the reviewers. Any product that may be evaluated in this article, or claim that may be made by its manufacturer, is not guaranteed or endorsed by the publisher.
